# Association of Lifestyle‐Induced Weight Loss With Gene Expression in Subcutaneous Adipose Tissue in Metabolic Syndrome

**DOI:** 10.1111/1753-0407.70083

**Published:** 2025-04-14

**Authors:** Silke Zimmermann, Kirsten Roomp, Hans‐Jonas Meyer, Akash Mathew, Manuel Florian Struck, Matthias Blüher, Hugo N. G. Martin, Maria Keller, Kathrin Landgraf, Antje Körner, Anne Hoffmann, Yvonne Böttcher, Kathleen Biemann, Adhideb Ghosh, Christian Wolfrum, Falko Noé, Berend Isermann, Jochen G. Schneider, Ronald Biemann

**Affiliations:** ^1^ Institute of Laboratory Medicine, Clinical Chemistry and Molecular Diagnostics University of Leipzig Leipzig Germany; ^2^ Luxembourg Centre for Systems Biomedicine (LCSB) University of Luxembourg Luxembourg Luxembourg; ^3^ Diagnostic and Interventional Radiology University of Leipzig Faculty of Medicine Leipzig Germany; ^4^ Department of Anesthesiology and Intensive Care Medicine University Hospital Leipzig Leipzig Germany; ^5^ Helmholtz Institute for Metabolic Obesity and Vascular Research (HI‐MAG) of the Helmholtz Zentrum München at the University of Leipzig and University Hospital Leipzig Leipzig Germany; ^6^ Medical Department III—Endocrinology, Nephrology, and Rheumatology Leipzig University Medical Center Leipzig Germany; ^7^ German Center for Child and Adolescent Health (DZKJ) Leipzig/Dresden Partner Site Leipzig Germany; ^8^ Center for Pediatric Research Leipzig (CPL), Hospital for Children & Adolescents University of Leipzig Leipzig Germany; ^9^ University of Oslo Institute of Clinical Medicine, Department of Clinical Molecular Biology, EpiGen Oslo Norway; ^10^ Medical Division, EpiGen Akershus University Hospital Lørenskog Norway; ^11^ Institute of Food, Nutrition and Health ETH Zurich Schwerzenbach Switzerland; ^12^ Department of Internal Medicine II Saarland University Medical Center at Homburg/Saar Homburg Germany; ^13^ Centre Hospitalier Emile Mayrisch Esch sur Alzette Luxembourg

**Keywords:** differentially expressed genes (DEGs), lifestyle‐induced weight loss (LIWL), metabolic syndrome (MetS), subcutaneous adipose tissue (SAT)

## Abstract

**Aims:**

Lifestyle‐induced weight loss (LIWL) is considered an effective therapy for the treatment of metabolic syndrome (MetS). The role of differentially expressed genes (DEGs) in adipose tissue function and in the success of LIWL in MetS is still unclear. We investigated the effect of 6 months of LIWL on transcriptional regulation in subcutaneous adipose tissue (SAT). Aiming to identify a LIWL‐associated “gene signature” in SAT, DEGs were fitted into a linear regression model.

**Materials and Methods:**

The study is embedded in a prospective, two‐arm, controlled, monocentric, randomized, 6‐month interventional trial in individuals with MetS following LIWL. The trial included 43 nonsmoking, nondiabetic men aged 45–55 years with MetS.

**Results:**

In total, we identified 642 DEGs in SAT after 6 months of LIWL. The identified DEGs were validated in two cross‐sectional cohorts analyzing SAT from individuals with and without obesity. Gene enrichment analysis of the DEGs revealed the strongest association with cholesterol metabolic processes. Accordingly, DEGs were correlated with the lipid parameters HDL cholesterol, LDL cholesterol, and triglycerides in corresponding serum samples. We identified 3 genes with an AUC of 0.963 (95% CI: 0.906–1.0) associated with a loss of more than 10% of initial body weight that was maintained for at least 12 months after LIWL, namely *SUMO3* (*Small ubiquitin‐related modifier 3*), *PRKG2* (*Protein Kinase CGMP‐Dependent 2*), and *ADAP2 (ArfGAP with Dual PH Domains 2*).

**Conclusion:**

In summary, we have identified DEGs in SAT after LIWL, which may play an important role in metabolic functions. In particular, altered gene expression in SAT may predict sustained weight loss.

## Graphical abstract

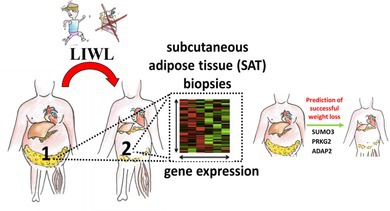

**Summary**

- Lifestyle‐induced weight loss (LIWL) significantly affects gene expression in subcutaneous adipose tissue (SAT), identifying 642 differentially expressed genes linked to cholesterol metabolism.
- The genes SUMO3, PRKG2, and ADAP2 emerged as potential biomarkers for predicting sustained weight loss of over 10% maintained for at least 12 months.
- These findings highlight the role of specific gene alterations in metabolic functions and emphasize the need for further studies in larger cohorts to validate these results.

## 1 Introduction

Metabolic syndrome (MetS) is a cluster of dysregulated metabolic traits, including insulin resistance, atherogenic dyslipidemia, central obesity, and hypertension [1]. According to the Centers for Disease Control and Prevention (CDC), the prevalence of MetS in the United States increased by more than 35% between the time the term MetS was introduced in the 1980s and 2018 [2, 3]. MetS is associated with an increased risk of developing both cardiovascular diseases (CVD) and type 2 diabetes (T2D). The pathophysiology of MetS involves several complex mechanisms that are not fully understood. Lifestyle and environmental factors, such as high calorie intake and physical inactivity, are strongly associated with obesity and insulin resistance and have been identified as major contributors to the development of MetS [4].

White adipose tissue (WAT) is the major fat storage depot and also serves as the largest endocrine organ secreting adipokines, hormones (e.g., leptin, adiponectin), peptides, and inflammatory cytokines, which play an important role in the pathophysiology of insulin resistance in MetS. As some adipokine levels correlate with increased cardiovascular risk and insulin resistance, several adipokines have been suggested as important factors linking obesity, CVD, and T2D via metabolic dysregulation.

As obesity and atherogenic dyslipidemia are hallmarks of the MetS, treatment guidelines recommend moderate weight loss of 5%–10% to achieve improvements in metabolic function and health outcomes [5]. It is well established that lifestyle‐induced weight loss (LIWL), based on behavioral therapy and calorie reduction, is an effective strategy for managing MetS and preventing disease progression [6, 7, 8]. Studies have demonstrated that individuals with obesity show changes in their metabolic profile after weight loss intervention [9].

Gene expression changes in adipose tissue have been implicated in the pathogenesis of metabolic disorders, providing insights into potential biomarkers and therapeutic targets.

Recent studies have illuminated gene expression alterations in adipose tissue that accompany metabolic diseases. For instance, it has been shown that patients with obesity exhibit significant gene expression changes in genes involved in inflammation, lipid metabolism, and insulin signaling pathways [10, 11, 12] . These changes are not only indicative of the disease state but also appear to contribute to the progression of metabolic dysfunction. In individuals with T2D, for instance, dysregulated expression of genes involved in glucose metabolism, such as those coding for glucose transporters and enzymes involved in glycolysis and gluconeogenesis, has been reported [13]. Moreover, the chronic inflammatory state observed in obesity is reflected in the upregulation of pro‐inflammatory cytokines and chemokines, which exacerbates insulin resistance and contributes to the pathophysiology of MetS [14].

Several candidate genes and molecular signatures have been proposed as predictive markers in long‐term metabolic success [15]. After a successful weight loss intervention, certain genes related to inflammation and insulin sensitivity tend to revert toward a healthier expression pattern, which correlates with improvements in insulin sensitivity and lipid profiles [16]. However, the persistence of these gene expression changes, and whether they can predict long‐term success in weight management and metabolic improvement, is still under debate [17]. In this context, identifying stable gene expression markers that can predict the likelihood of achieving and maintaining a healthy weight and metabolic profile after weight loss interventions is of great clinical importance.

Although randomized controlled trials have been conducted, only a few studies investigated changes in adipose tissue gene expression signatures following LIWL in a longitudinal setting [18, 19]. To identify genes that are associated with LIWL in MetS, we analyzed gene expression in subcutaneous adipose tissue (SAT) samples before and after 6 months of LIWL in a controlled intervention study. The aims of this study were (i) to determine whether gene expression in adipose tissue changes after weight loss, (ii) whether specific gene changes are associated with improved metabolic parameters, and (iii) whether a LIWL‐associated gene signature is able to predict successful weight loss.

## 2 Materials and Methods

### 2.1 Research Design and Study Population

The study is embedded in a prospective, two‐arm, controlled, monocentric, randomized, 6‐month interventional trial aimed to identify changes in gene expression in SAT in individuals with MetS following LIWL as described previously [20, 21, 22]. The trial was registered at the German Clinical Trials Register (ICTRP Trial Number: U1111‐1158‐3672). The trial included nonsmoking, nondiabetic men aged between 45 and 55 years with MetS, as defined by the consensus definition in 2009 [1]. Three out of the following five criteria needed to be met: abdominal obesity (waist circumference > 102 cm or BMI > 30 kg/m^2^); fasting triglyceride (TG) concentration ≥ 1.7 mmol/L (or pharmaceutical intervention); high‐density lipoprotein (HDL) cholesterol < 1.00 mmol/L; fasting plasma glucose (FPG) ≥ 5.6 mmol/L (or pharmaceutical intervention); and blood pressure ≥ 130/85 mmHg or treatment for hypertension. Participants were recruited by an advertisement in a regional newspaper. Repeated (before and after LIWL) blood samples and SAT biopsies were collected at the Institute of Clinical Chemistry and Pathobiochemistry, Otto‐von‐Guericke University (OvGU), Magdeburg, Germany.

30 participants in the control arm and 33 participants in the treatment arm completed the study. During the follow‐up, participants initially assigned to the control arm were offered enrolment in the treatment arm and received exactly the same weight loss intervention as participants of the initial treatment group. Only paired sample sets with high RNA quality were selected for gene expression profiling. In total, 43 subjects with paired adipose tissue samples and weight loss > 5% were included in the transcriptome data analysis: 24 subjects from the treatment arm and 19 subjects from the control arm who subsequently underwent LIWL during the follow‐up period (Supporting Information Figure 1). Thus, all individuals included in the study underwent LIWL, and SAT biopsies were obtained before and after LIWL in all.

### 2.2 Clinical and Laboratory Parameters

All blood samples were collected in the morning (8 am to 9 am) from the antecubital vein after a 12‐h overnight fast. All laboratory measurements were performed at the Institute of Clinical Chemistry and Pathobiochemistry, OvGU, Magdeburg, Germany. FPG, TGs, LDL cholesterol, and HDL cholesterol were analyzed by commercial enzymatic methods using a random‐access analyzer (Modular, Roche Diagnostics, Mannheim, Germany).

### 2.3 Transcriptome Profiling

Processing of RNA and generation of transcriptomic data was performed at the European Molecular Biology Laboratory (EMBL, Germany). A total of 300 ng of total RNA was used to prepare labeled and purified sense‐strand cDNA (Ambion WT Expression Kit, Thermo Fisher Scientific, USA). Reactions from all samples yielded sufficient cDNA for subsequent microarray analysis. Samples were hybridized to Affymetrix GeneChip Human Gene 2.0 ST transcript arrays (Thermo Fisher Scientific, USA) covering more than 30,000 coding transcripts, including alternatively spliced variants, and more than 11,000 long intergenic noncoding transcripts. Hybridization was carried out according to the standard protocol provided by the manufacturer. Raw data were preprocessed with the Robust Multiarray Average algorithm (RMA; convolution background correction, quantile normalization and summarization based on the median polish algorithm) [23]. Differentially expressed genes (DEGs) were identified using the limma package [24] in R programming language, which fits a linear model for each gene based on the given series of arrays, creates an appropriate contrast matrix to perform all pairwise comparisons, computes estimated coefficients and standard errors for a given set of contrasts, and computes moderated t‐statistics and log‐odds of differential expression by empirical Bayes. Correction for multiple testing was performed using the Benjamini‐Hochberg method to control the false discovery rate [25]. The principal components analysis (PCA) plot was generated using the affycoretools package [26].

### 2.4 Network and Enrichment Analysis

The network analysis was performed with all identified significantly DEGs as input, using STRING 11.0 [27]. The minimum required interaction score selected was that of the highest confidence (0.900) and all available activation interaction sources were included (text‐mining, experiments, databases, co‐expression, neighborhood, gene fusion, co‐occurrence). The network data from STRING was exported to Cytoscape [28] to visually add the relative logFC changes to each node in the network: green indicates the gene was downregulated after LIWL, red that the gene was upregulated after LIWL, with the color intensity indicating the level of logFC. Completely disconnected nodes in the network were excluded from the figure that was generated. For the gene enrichment analysis (http://bioinformatics.sdstate.edu/go/), we specifically looked at Gene Ontology (GO) in order to determine which genes were statistically overrepresented in biological processes [29, 30].

### 2.5 Prediction of Successful Weight Loss in LIWL

Aiming to identify a weight loss associated “gene signature” in the SAT, identified DEGs in SAT following LIWL were fit into a regression model in order to elaborate whether they can predict successful weight loss. According to the definition of Hill and Wing, successful weight loss is achieved when initial weight loss is greater than 10% of body weight and can be maintained for at least 12 months [31]. The predictors obtained in the LIWL cohort were validated in the independent two‐step bariatric surgery cohort (BSC). The two‐step BSC, which is also part of LOBB, consists of individuals with morbid obesity (BMI > 40 kg/m^2^) who underwent a two‐step bariatric surgery approach, primarily involving sleeve gastrectomy as the first step, followed by Roux‐en‐Y gastric bypass as the second step, as previously reported [32]. Only those who lost more than 10% of their body weight between the two surgeries were included in the analysis, resulting in a total of 61 participants (33% male; first step: age: 46 ± 10.1 years, BMI: 54.3 ± 9.3 kg/m^2^; second step: age: 47 ± 10.2 years, BMI: 40.6 ± 7.2 kg/m^2^). On average, the patients lost 39.4 ± 20 kg between the two surgeries.

### 2.6 Validation Cohorts

The results of this longitudinal LIWL study were compared with gene expression of SAT samples data from two independent cohorts: (i) A cross‐sectional “Leipzig Adipose Tissue Childhood Cohort” (LATC) [33] and (ii) a cohort of adults with or without obesity (OA) [34, 35].

Gene expression data from SAT samples obtained from 317 children of the LATC cohort was derived from a previously performed analyses [35]. The study was approved by the local ethics committee (Reg. No: 265‐08, 265‐08‐ff, University of Leipzig; NCT02208141). The SAT samples of the LATC cohort were obtained during elective surgery (i.e., orthopedic surgery, herniotomy or orchidopexy, abdominal surgery, back surgery, and mammary reduction surgery).

The OA cohort is an integral part of the Leipzig Obesity Biobank (LOBB; https://www.helmholtz‐munich.de/en/hi‐mag/cohort/leipzig‐obesity‐bio‐bank‐lobb) and has been previously reported by Keller et al. [34] This cohort comprises 25 adults without obesity (36% male, age: 64 ± 11.4 years, BMI: 23.4 ± 1.9 kg/m^2^) and 38 subjects with obesity (18% male, age: 45 ± 13.1 years, BMI: 43.4 ± 11.7 kg/m^2^), selected due to the availability of mRNA expression data. All participants underwent open abdominal surgeries, including cholecystectomy and weight reduction procedures.

SAT samples from LOBB were collected during surgeries as previously described [36, 37]. All laboratory measurements were performed concurrently [38, 39].

### 2.7 Transcription Profiling of the OA Cohort

Genome wide expression profiling was performed using Illumina human HT‐12 expression chips. RNA integrity and concentration were examined using Agilent 2100 Bioanalyzer (Agilent Technologies, Palo Alto, CA, USA). Raw data were preprocessed using the limma [24] (v3.42.69) and beadarray [40] (v2.50.0) R packages. Additionally, the arrayQualityMetrics package [41] (v3.42.68) was employed for data quality control. DEGs between individuals with or without obesity were identified using the linear models for microarray data (LIMMA) method implemented in the limma R package. To enhance the signal‐to‐noise ratio, array weights were incorporated into the linear model. Only DEGs with an adj. *p* < 0.05 were deemed statistically significant.

### 2.8 Bulk RNA Sequencing and Analysis of the Bariatric Surgery Cohort (BSC)

Total RNA was extracted from SAT, and ribosomal RNA‐depleted RNA sequencing (RNA seq) data were generated following the SMARTseq protocol [42, 43]. All libraries were sequenced as single‐end reads on a Novaseq 6000 instrument (Illumina, San Diego, CA, USA) at the Functional Genomics Center Zurich, Switzerland. Processing of the RNAseq data is described in detail [32]. The data were normalized using a weighted trimmed mean (TMM) of the log expression ratios. The data were adjusted for age and gender using limma v3.56.2 [44]. To address the impact of in vitro RNA degradation, we adjusted normalized counts by utilizing transcript integrity numbers (TINs) estimated with RSeQC v4.0.0 [24]. Analyses were carried out in R v4.3.1 (www.R‐project.org).

### 2.9 Statistical Analysis

Data are given as median and interquartile range (IQR). Paired samples were analyzed by Wilcoxon Signed‐Rank Test. Calculations were performed using the IBM SPSS Statistics, version 29.0 (IBM Corporation, Armonk, NY, USA). Results were considered statistically significant at *p* < 0.05. The data analysis was done using graph pad prism version 8.0. Anthropometric and biomedical characteristics (untransformed and log transformed) were tested for normality (normal distribution) using the Shapiro–Wilk test. Subsequently, these medical characteristics and the previously identified DEGs were tested for association using the Spearman’ rank coefficient of correlation, which is a nonparametric measure of the statistical dependence of ranking between two variables. Statistically significant results, where the Spearman Coefficient rho had a *p* value of less than 0.05, were reported both in graphical and tabular format.

For the prediction model, we used a logistic regression model due to its reduced small number of input features. Prior to fitting the gene changes and the dichotomous variable (successful versus nonsuccessful weight loss) to the logistic regression model, we performed a correlation analysis using Python (version 3.6) for Windows. In order to account for nonnormal features, we opted for Spearman correlation. We removed correlating features with correlation larger than 0.7 in order to avoid weakening of the regression model by loading variables which correlate. Further, we only used the 642 identified genes (significant DEGs). The SPSS modeler was used in order to further reduce feature count and ranking of features of the selection model in order to extract the key features for the model. Based on the ranking results, we fitted the features in forward regression analysis. We selected the optimal combination (feature signature), which was able to predict the success of weight loss of the participants. The receiver operating characteristic (ROC) curve was used to evaluate and show the performance of the model for discrimination between participants with successful versus nonsuccessful weight loss as defined by Hill et al. [31].

## 3 Results

We analyzed changes in parameters characteristic of the MetS, i.e., BMI, fasting plasma glucose, HDL cholesterol, systolic and diastolic blood pressure, and triglyceride levels before and after LIWL and found an improvement in their metabolic status (Supporting Information Figure 1, Table 1). To this end, we observed a reduction in BMI (−13%), fasting plasma glucose (−7%), blood pressure (−7.1% systolic; −5.6% diastolic) and triglycerides (−35.7%) as well as an increase in HDL cholesterol (+16.9%). The median body weight loss after 6‐month LIWL was 13.2 kg, which is congruent with our findings in the original cohort that included only participants of the treatment arm [9, 21].

**TABLE 1:** Clinical parameters of the study population before and after lifestyle‐induced weight loss (LIWL).

	Before LIWL	After LIWL	*p*
Age (years)	48.0 ± 6		—
BMI (kg/m^2^)	33.13 ± 4.24	28.83 ± 4.1	< 0.001
FPG (mmol/L)	6.04 ± 0.72	5.62 ± 0.48	< 0.001
HDL cholesterol (mmol/L)	1.24 ± 0.38	1.45 ± 0.44	< 0.001
RR systolic (mmHg)	140 ± 20	130 ± 20*	< 0.001
RR diastolic (mmHg)	89 ± 13	84 ± 10*	0.002
Triglycerides (mmol/L)	1.99 ± 1.9	1.28 ± 0.85*	< 0.001

*Note:* Data are presented as the median ± IQR. Wilcoxon signed‐rank test was used to analyze changes between paired samples before and after LIWL, *n* = 43 participants.

Abbreviations: BMI = body mass index, FPG = fasting plasma glucose, HDL = high density lipoprotein, RR = blood pressure.

* *p* < 0.05.

### 3.1 Transcriptomic Profiling

**FIGURE 1:**
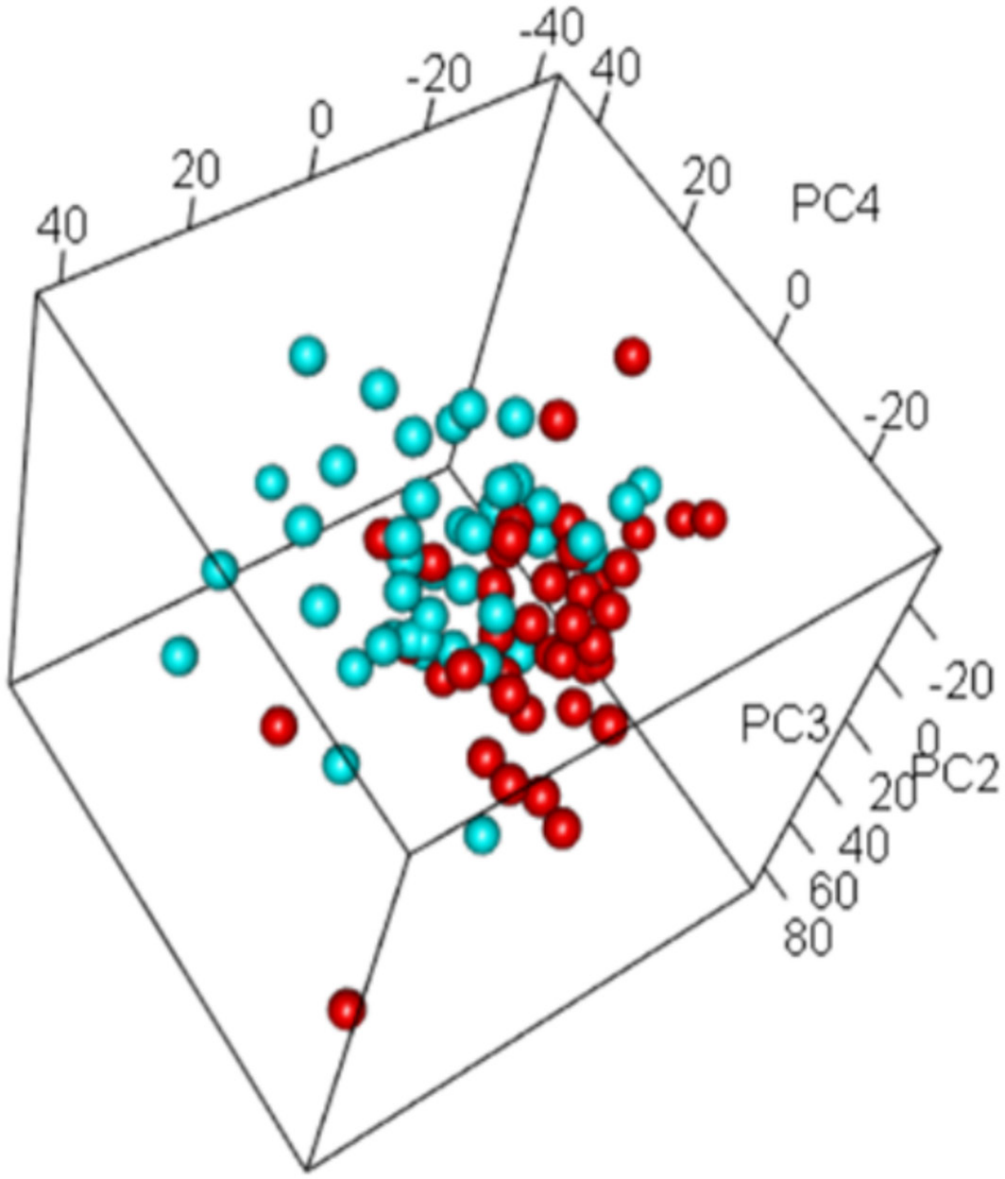
Three‐dimensional principal component analysis (PCA) of adipose tissue samples before and after lifestyle‐induced weight loss. The PCA plot was used to visualize the variance in gene expression between corresponding subcutaneous adipose tissue samples before (red spheres, *n* = 43) and after weight loss (blue spheres, *n* = 43). For optimal visual separation, we used a 3D PCA plotting approach, showing the PC2, PC3, and PC4‐axes (black lines) capturing the linear combinations comprising the second, third and fourth largest percentages of variance, respectively. The proportion of variance explained by PC2 = 0.0591, PC3 = 0.0562, and PC4 = 0.0347. The PCA was generated using the affycoretools package MacDonald [26].

Principal component analysis (PCA) was used to visualize differences in gene expression before and after LIWL. The PCA yielded a good separation between gene expression results before and after LIWL (Figure 1), which prompted us to investigate the differences in detail.

To identify the impact of LIWL on gene expression in SAT, we analyzed DEGs in paired SAT samples before and after LIWL by microarray analysis. The analysis revealed 657 DEG probe identifiers, which survived multiple testing (FDR‐adjusted *p* < 0.05), which represent 642 unique genes.

The top five upregulated genes were storkhead box 1 (STOX1), myosin regulatory light chain interacting protein (MYLIP), cholesteryl ester transfer protein (CETP), aminoadipate semialdehyde synthase (AASS) and apolipoprotein E (APOE), while quinone 1 (NQO1), colony stimulating factor 1 receptor (CSF1R), B cell linker (BLNK), synuclein gamma (SNCG) and purinergic receptor p2x 6 pseudogene (P2RX6) were the top 5 downregulated genes. The top 20 DEGs are listed in Table 2.

**TABLE 2:** Top 20 differentially expressed genes (ranked by *p* value) in subcutaneous adipose tissue following lifestyle‐induced weight loss.

AffyID	logFC	AveExpr	adj.P.Val	annot
17 005 077	0.35	8.82	9.63E‐05	MYLIP
16 819 336	0.81	6.62	9.63E‐05	CETP
17 062 280	0.37	8.17	2.26E‐04	AASS
16 827 679	−0.63	10.22	2.52E‐04	NQO1
17 001 545	−0.41	9.43	5.19E‐04	CSF1R
17 051 914	0.33	5.27	5.19E‐04	AGBL3
17 059 613	0.24	5.14	5.19E‐04	CTB‐111F10.1
16 712 168	0.22	6.22	5.19E‐04	CUBN
16 716 918	−0.41	6.03	5.19E‐04	BLNK
16 706 896	−0.36	8.12	5.19E‐04	SNCG
17 075 082	−0.25	9.92	5.93E‐04	ASAH1
16 705 439	0.89	6.12	5.93E‐04	STOX1
16 712 879	0.30	7.64	5.93E‐04	SVIL
16 863 115	0.46	5.51	6.13E‐04	APOE
16 852 025	0.41	5.67	6.13E‐04	FHOD3
16 927 491	−0.34	6.89	6.87E‐04	P2RX6
17 103 951	0.29	6.37	6.87E‐04	TSPYL2
16 754 729	0.34	8.74	7.24E‐04	ACSS3
16 752 190	−0.29	9.15	8.45E‐04	RDH5
17 106 624	−0.28	5.50	9.40E‐04	RP4‐755D9.1

Abbreviations: *adj.P.Val*: the *p* value adjusted for multiple testing; *AffyId:* the Affymetrix probe identifier; *annot*: the gene name as per HGNC; *AveExpr:* average log2‐expression for the probe over all arrays and channels; *logFC:* the log fold‐change between paired samples before and after lifestyle‐induced weight loss.

**FIGURE 2:**
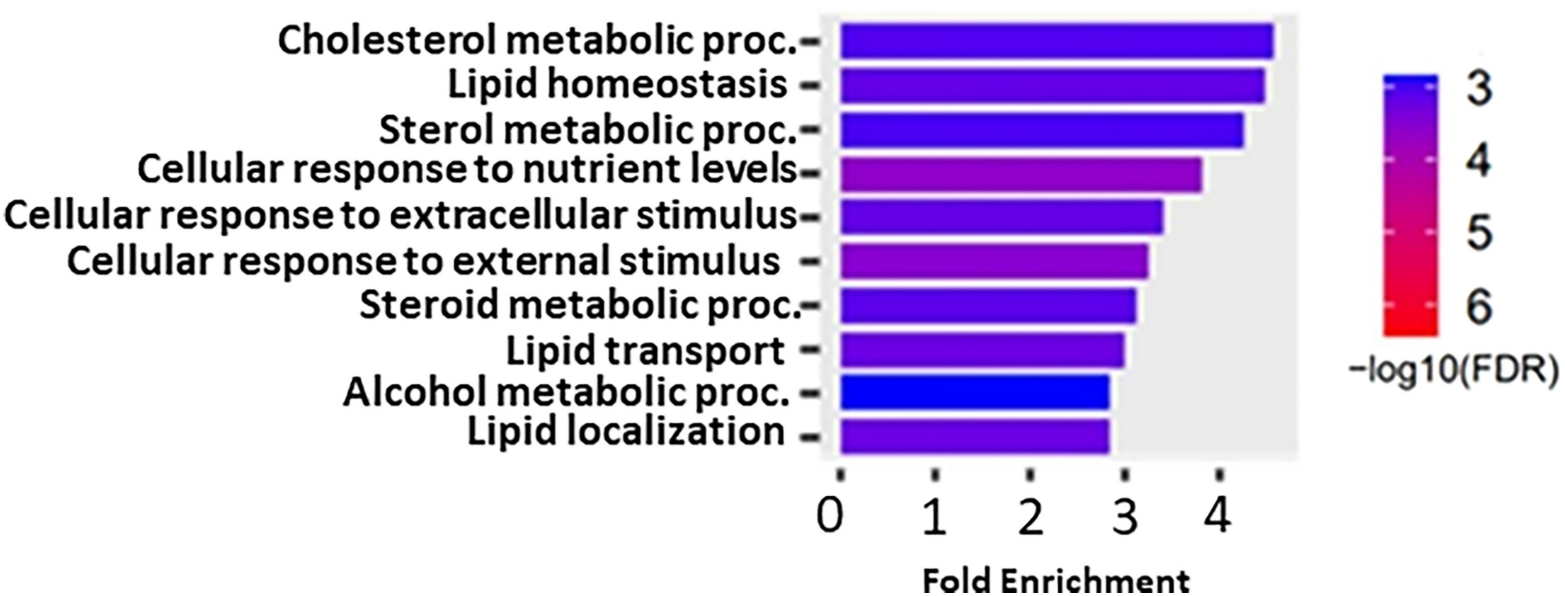
Enrichment analysis for biological processes of DEGs in MetS participants undergoing LIWL. The 10 most enriched biological processes are shown. DEGs = differentially expressed genes, FDR = false discovery rate, LIWL = lifestyle‐induced weight loss, MetS = metabolic syndrome.

To identify pathways associated with altered gene expression in SAT following LIWL, gene enrichment analysis employing shinyGO [45]. All identified 642 DEGs were included in the analysis. The genes that were differentially expressed in the SAT following LIWL showed the highest association with cholesterol metabolic processes in the GEO biological process annotation (Figure 2). Interestingly, 6 out of the top 20 biological processes were related to lipid and cholesterol metabolism.

**FIGURE 3:**
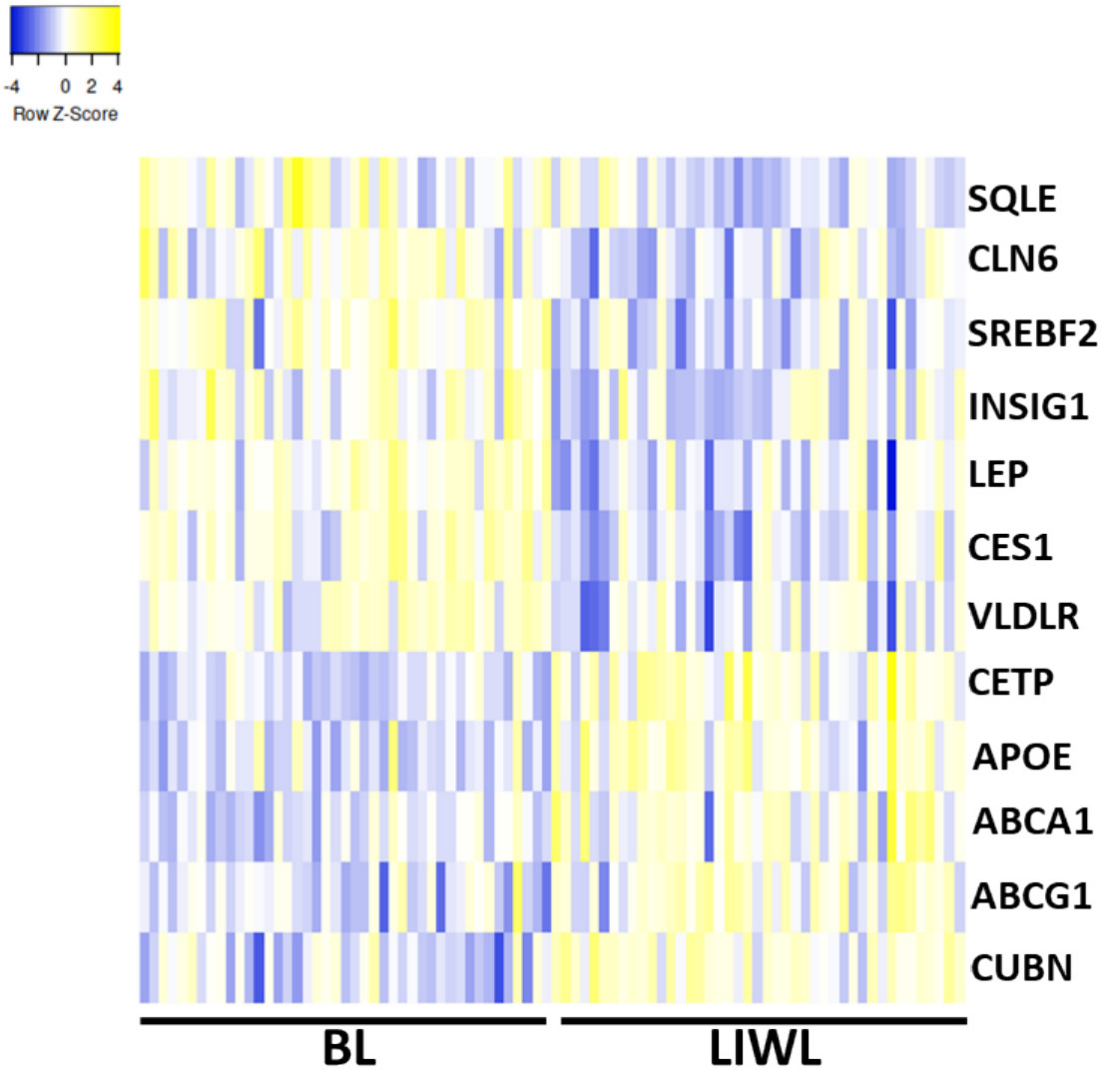
Heatmap showing the expression of genes enriched in the biological process “cholesterol metabolic process” before (BL) and after lifestyle‐induced weight loss (LIWL) in subcutaneous adipose tissue. ABCA1 = ATP‐binding cassette transporter, ABCG1 = ATP‐binding cassette sub‐family G member 1, APOE = apolipoprotein E, BL = baseline, Blue = negatively changed genes, CES1 = carboxylesterase 1, CETP = cholesteryl ester transfer protein, CLN6 = ceroid‐lipofuscinosis neuronal protein 6, CUBN = cubilin, INSIG1 = insulin induced gene 1, LEP = leptin, LIWL = lifestyle‐induced weight loss, SQLE = squalene epoxidase, SREBF2 = sterol regulatory element binding transcription factor 2, VLDLR = very‐low‐density‐lipoprotein receptor, yellow = positively changed genes.

Regarding the genes associated with the cholesterol metabolic process, the genes CETP, APOE, ATP‐binding cassette transporter (ABCA1), ATP‐binding cassette sub‐family G member 1 (ABCG1) and cubilin (CUBN) were upregulated following LIWL, while ceroid‐lipofuscinosis neuronal protein 6 (CLN6), carboxylesterase 1 (CES1), insulin‐induced gene 1 (INSIG1), leptin (LEP), squalene epoxidase (SQLE), sterol regulatory element binding transcription factor 2 (SREBF2) and very‐low‐density‐lipoprotein receptor (VLDLR) were downregulated after LIWL (Figure 3). Notably, CES1 and LEP were negatively correlated to changes in HDL cholesterol following LIWL and positively correlated to triglyceride changes, whereas CETP, APOE, and ABCA1 showed negative correlations to BMI changes in LIWL. ABCG1, CUBN, CLN6, INSIG1, SQLE, and VLDLR were not correlated to any changes in MetS parameters.

**FIGURE 4:**
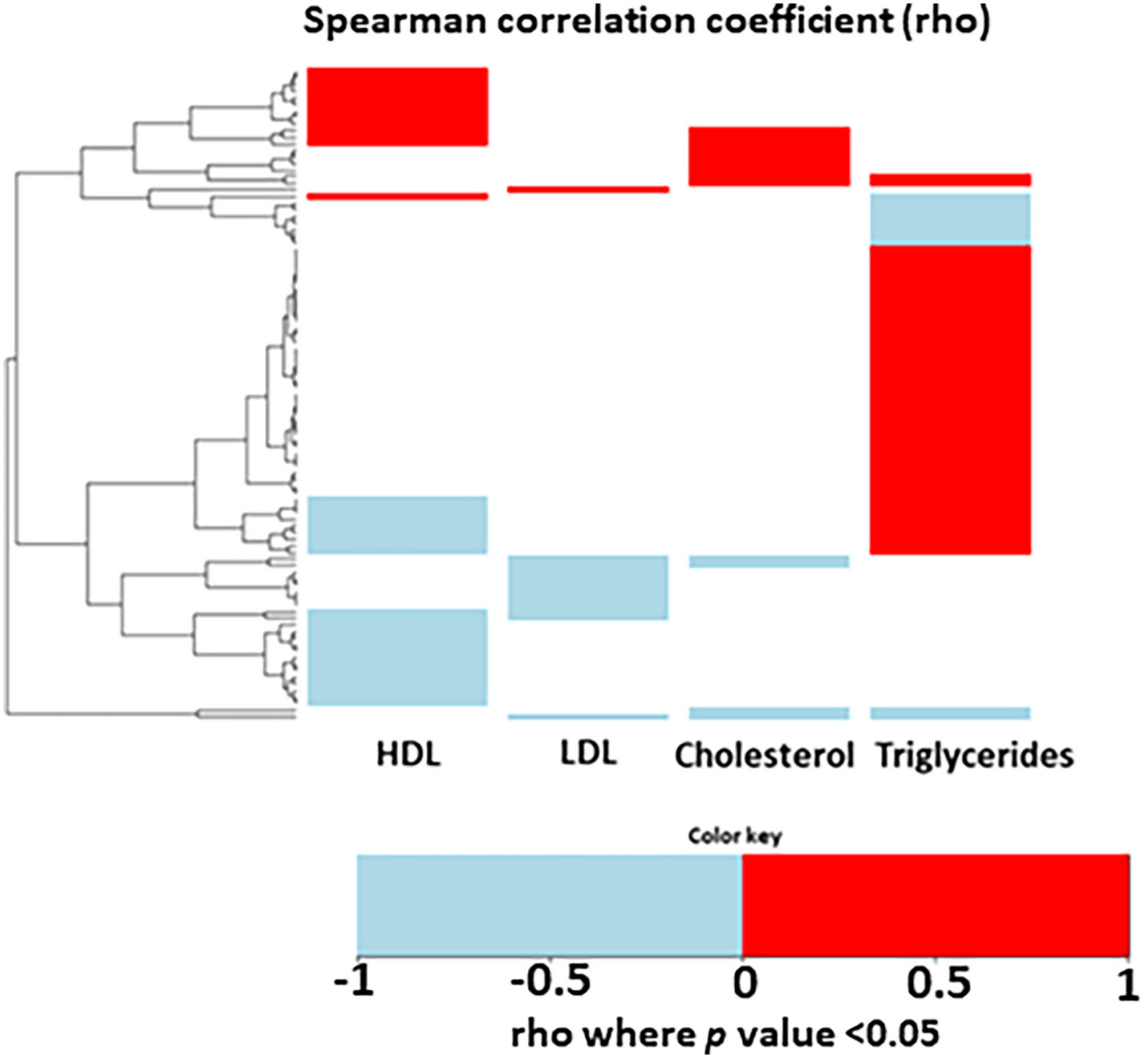
Correlation of differentially expressed genes (DEGs) with HDL cholesterol, LDL cholesterol, total cholesterol or triglycerides in lifestyle‐induced weight loss (LIWL). Heatmap representation of Spearman's correlation coefficient analysis for all DEGs with a Spearman's correlation coefficient *p* value < 0.05 (comprising 242 DEGs) for lipid and cholesterol related parameters in the cohort: Triglycerides, LDL cholesterol (LDL), HDL cholesterol (HDL), total cholesterol (Cholesterol). Positive rhos (red) indicate a positive relationship between the two variables, negative rhos (blue) indicate that the variables are inversely related. The dendrogram groups genes with a more similar significant correlation pattern together. The values in tabular format can be found in Supporting Information Table 2.

To evaluate whether changes in gene expression correlate with changes in lipid and cholesterol metabolism, we analyzed the correlation of gene changes in SAT with changes in triglycerides, LDL cholesterol, HDL cholesterol, and total cholesterol levels. We identified a total of 242 genes that correlated with changes in triglyceride, LDL cholesterol, HDL cholesterol, and total cholesterol levels. Most of the genes correlated to changes in triglycerides (Supporting Information Table 1, Figure 4, 23.5% of the DEGs); 14.5% of the DEGs correlated with changes in HDL cholesterol, whereas only 5% and 5.4% of the DEGs correlated with changes in LDL cholesterol and changes in total cholesterol, respectively. Notably, the overall highest correlation was shown between CES1 and triglyceride changes (*r* = 0.51) or HDL cholesterol changes (*r* = −0.32).

### 3.2 Prediction of Successful Weight Loss

**FIGURE 5:**
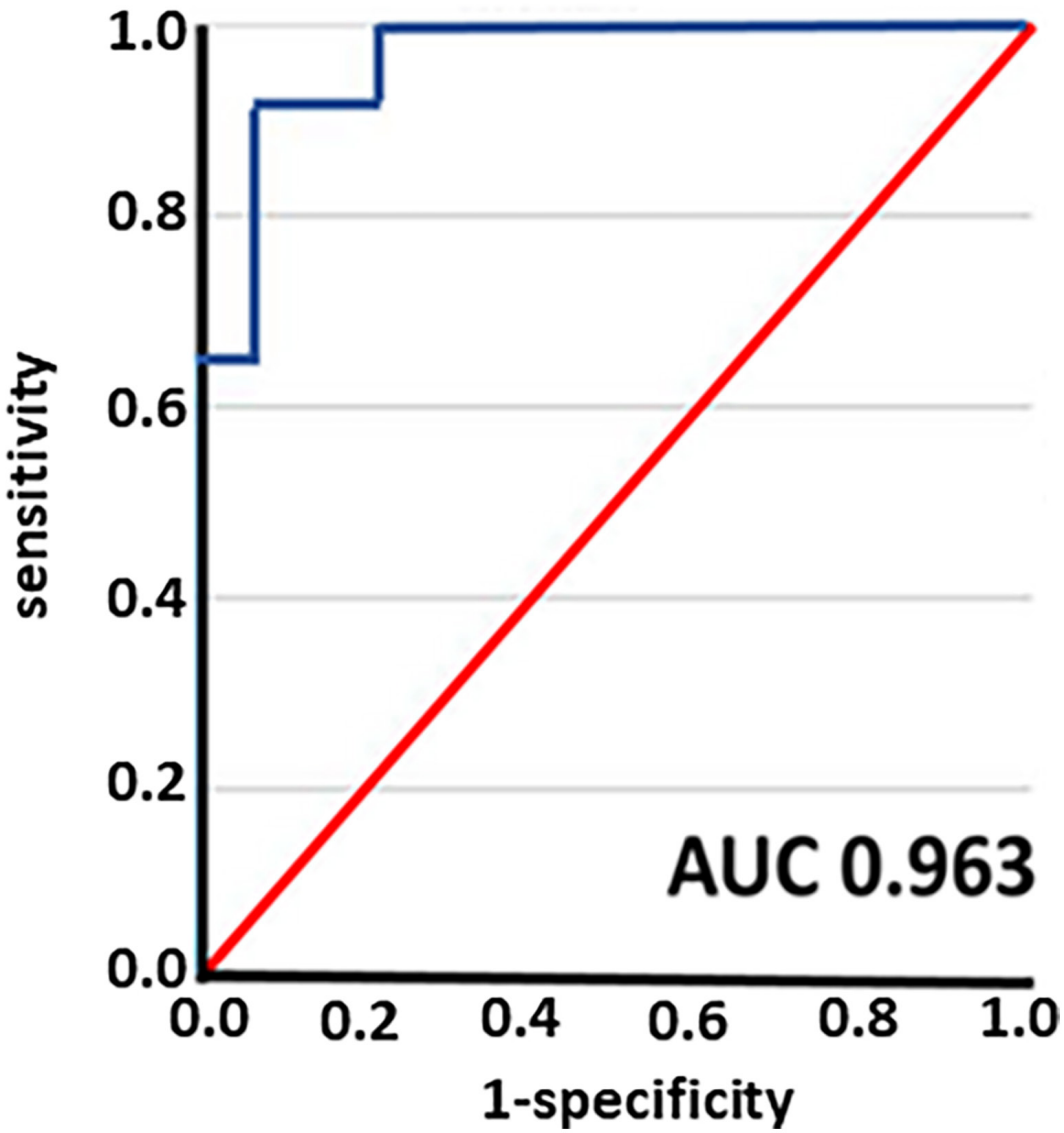
Combination of weight loss‐associated genes SUMO3, PRKG2 and ADAP2 predict long term weight loss. ROC analysis was performed to predict successful weight loss in LIWL. Predicted probability (blue line) and reference line (red). AUC was 0.963 (95% CI: 0.906–1.0). AUC = area under the curve.

To identify a “gene signature” associated with LIWL in the SAT, we next fitted identified DEGs into a regression model in order to elaborate whether the identified genes may predict successful weight loss. Out of 642 identified DEGs, we identified three genes that predicted successfully the weight loss of greater than 10% that was sustained for at least 12 months with an AUC of 0.963 (95% CI: 0.906–1.0, Figure 5a), namely SUMO3 (Small ubiquitin‐related modifier 3), PRKG2 (Protein Kinase cGMP‐Dependent 2) and ADAP2 (ArfGAP with Dual PH Domains 2). To avoid overfitting of the model with a relatively small sample size, we only used 3 predictors. Notably, the identified genes correlate with changes in fasting plasma glucose (ADAP2, *r* = 0.30), in HDL cholesterol changes (SUMO3, *r* = 0.38) and systolic blood pressure (PRKG2, *r* = 0.32) in LIWL. Due to the small sample size, we could not build an independent validation model in our cohort.

For the validation of our findings, we used an independent external cohort, consisting of 65 individuals who had undergone bariatric surgery (bariatric surgery cohort, BSC). Despite the different weight loss methods in both studies (LIWL vs. BSC), the same identified genes predicted successful weight loss in the validation cohort with an AUC of 0.655 (95% CI: 0.504–0.841, Supporting Information Figure 2). These results suggest that weight loss‐associated changes in gene expression of SUMO3, PRKG2, and ADAP2 in SAT are associated with long‐term weight loss independently of the different methods to induce weight loss.

### 3.3 Network and Enrichment Analysis

We next used the STRING gene set enrichment analysis to predict possible interactions between identified DEGs [27]. We entered identified 642 DEGs into STRING 11.0 for network analysis. Of these, 520 are included in the STRING database and are used to generate a network where nodes represent the gene and where edges are indicative of both functional and physical interactions. Since nodes can share multiple edges, STRING network enrichment identified a significant gene enrichment network with 1410 edges, an average node degree of 5.42, and an average local clustering coefficient of 0.368. All nodes with at least one edge identified in STRING were uploaded into Cytoscape in order to show both negative and positive log fold changes in gene expression (Supporting Information Figure 3).

Genes that were found in GEO BP analysis “cholesterol metabolic process” and the top five up‐ or downregulated genes are visualized within the network with a yellow or blue circles, respectively (Supporting Information Figure 3). We thereby provide evidence of possible functional interaction in the genes found to be differentially expressed. Especially the genes relating to cholesterol metabolic process (yellow) show a strong clustering.

### 3.4 Validation With Two Cross‐Sectional Studies

We next attempted to validate the results yielded from the LIWL cohort with gene expression results in SAT of a cohort of children [35] (LATC) with and without obesity and a cohort of male adult patients with and without obesity (OA) [34]. In the LATC cohort and in the OA cohort, a total of 5705 and 6 DEGs were identified between children with or without obesity or male adult patients.

The DEG analysis of SAT in the LIWL cohort and the two other cohorts revealed 39.6% overlap of the DEGs in the LIWL cohort with the LATC cohort, and all identified DEGs in the OA cohort overlapped with the LIWL and LATC cohort (Supporting Information Figure 4): The genes glycerol‐3‐phosphate dehydrogenase 1 like (GPD1L), gasdermin B (GSDMB) and molybdenum cofactor synthesis 1 (MOCS1) were upregulated after LIWL, and their expression was also increased in SAT of individuals with or without obesity of the LATC and OA cohort. CD248, which has been recently linked with insulin metabolism [46], NAD(P)H quinone dehydrogenase 1 (NQO1) and tenomodulin (TNMD) were downregulated following LIWL, and their expression was also reduced in SAT of individuals with or without obesity of the LATC and OA cohort. Of note, all six overlapping genes were found to be regulated in the same direction among the three cohorts (Supporting Information Figure 5), indicating common regulation in all three studies.

## 4 Discussion

LIWL is an efficient method to ameliorate metabolic changes associated with MetS [6, 7, 9, 47]. The aim of this study was to identify LIWL‐associated changes in gene expression in SAT and to a reveal LIWL‐associated gene signatures. Analysis of paired tissue samples from individuals with MetS before and after LIWL identified 642 DEGs. We used gene set enrichment analysis to identify biological pathways that are regulated in SAT following LIWL. Of note, six out of the first 10 biological processes were related to lipid and cholesterol metabolism. Out of the 12 genes related to “cholesterol metabolism,” CES1 and LEP were correlated to changes in HDL cholesterol and triglyceride changes, whereas CETP, APOE, and ABCA1 showed negative correlations to BMI changes in LIWL, suggesting that cholesterol metabolism in SAT might be associated with plasma cholesterol changes. Data are visualized in STRING analysis showing associations between identified DEGs. For some of the most up‐ or downregulated DEGs, namely NQOI, GPD1L, and TNMD, the link to obesity, weight loss, or diabetes mellitus is described [48, 49, 50].

In order to validate gene expression results of the current LIWL study, we compared the results of two cross‐sectional cohorts, including one large cohort of children with or without obesity [35] (LATC) and a cohort of adult male patients with or without obesity [34] (OA). Despite methodological and study design‐related differences, the DEG analysis of SAT in the current study and the two other cohorts revealed that 39.6% of DEGs in the LIWL cohort overlapped with the LATC cohort, and all identified DEGs in the OA cohort showed overlap with the LIWL and the LATC cohort. Reassuringly, all six genes were found to be regulated in the same direction between individuals with or without obesity. This indicates a common gene signature in adipose tissue in individuals without obesity compared with individuals with obesity. The genes glycerol‐3‐phosphate dehydrogenase 1 like (GPD1L), gasdermin B (GSDMB) and molybdenum cofactor synthesis 1 (MOCS1) were upregulated after LIWL, and expression was increased in SAT of individuals with or without obesity of the LATC and OA cohorts. CD248, which has been recently linked with insulin metabolism [46], NAD(P)H quinone dehydrogenase 1 (NQO1) and tenomodulin (TNMD) were downregulated following LIWL, and expression was reduced in SAT of individuals with or without obesity of the LATC and OA cohorts. GPD1L was already found to be upregulated during weight loss and weight maintenance induced by low‐calorie diet (LCD), while downregulated during weight gain induced by high‐fat diet (HFD) [49].

*GPD1L* has been shown to be an important causal candidate gene for obesity‐associated complications in a cohort of humans undergoing weight loss intervention in gene expression profiling in human abdominal SAT [49]. Consistently, He et al. also identified *GPD1L* to be negatively correlated with insulin resistance.

Gasdermin B has been linked to the transformation of steatosis to steatohepatitis by controlling interleukin‐1β release, but so far no expression studies and mechanisms have been investigated [51].

The pericyte specific transmembrane receptor CD248 has been shown to be elevated in adipocytes of insulin‐resistant individuals with obesity. Interestingly, loss of CD248 in mice is linked to improved metabolic health via improved insulin sensitivity and reduced inflammation in adipose tissue [52]. CD248 has been shown to directly interact with the insulin receptor, blocking binding to insulin and promoting insulin resistance in mice [53]. In this regard, it is also of interest that hyperinsulinemia is an early sign of metabolic impairment even in young children with obesity [54]. Furthermore, the induction of CD248 has been identified to increase maladaptive unfolded protein response (UPR) signaling and inflammation [46]. Results of the current study show that LIWL is associated with a reduction of CD248 in SAT, which indicates improved insulin sensitivity and reduced SAT inflammation following LIWL. Accordingly, gene ontology analyses showed that high CD248 expression relates to pro‐inflammatory pathways, indicating its role as a sensor to hypoxia and cytokine release in SAT [52].

The expression of NAD(P)H quinone dehydrogenase 1 is high in adipose tissue and has been shown to be reduced following weight loss [55]. Interestingly, we found a high correlation of BMI reduction in LIWL with reduction of NQO1, fitting to the observation that NQO1 is higher expressed in larger adipocytes [55]. Finally, these findings implicate a role of NQO1 in the metabolic complications of obesity.

The TNMD gene is primarily expressed in SAT of subjects with obesity as compared to subjects without obesity [50]. Saiki et al. could reveal that TNMD is not only reduced in lean subjects but can also be reduced in SAT in diet‐induced weight loss, congruent with our findings. Further studies are required in order to decipher its potential role in adipose tissue function.

MOCS1 expression has not been associated with obesity or MetS so far. Further studies are necessary to investigate the role of MOCS1 in adipose tissue.

Aiming to develop personalized genetic markers for successful weight loss, we have identified a distinctive “gene signature” associated with weight loss in SAT. The significant DEG changes after LIWL were fit into a regression model in order to elaborate whether they can predict successful weight loss. According to Hill and Wing, successful weight loss is achieved when initial weight loss is greater than 10% of body weight and can be maintained for at least 12 months [31]. We identified three genes that predict successful weight loss with an AUC of 0.963 (95% CI: 0.906–1.0), namely SUMO3 (Small ubiquitin‐related modifier 3), PRKG2 (Protein Kinase CGMP‐Dependent 2) and ADAP2 (ArfGAP with Dual PH Domains 2). Whereas ADAP2, which binds beta tubulin and increases the stability of microtubule, and PRKG2, which is a crucial regulator of intestinal secretion and bone growth, have not been associated with weight loss or prediction models in metabolic disease settings, SUMO3 has previously been proposed to improve weight loss prediction models [56]. Interestingly, the identified genes correlate with changes in fasting plasma glucose (ADAP2, *r* = 0.30), HDL cholesterol changes (SUMO3, *r* = 0.38) and systolic blood pressure (PRKG2, *r* = 0.32) in LIWL. Another interesting question is whether changes in gene signatures in adipose tissue are specific for different weight loss programs. Furthermore, precision medicine aims to characterize each patient based on various diagnostic parameters. It would be interesting to design “patient‐specific signatures” based on laboratory parameters, imaging parameters, and clinical parameters rather than using gene signatures of the SAT, the latter being difficult to obtain. Due to the small sample size, we were unable to construct an independent validation model within the same cohort to confirm the weight loss‐associated “gene signature” results. However, we aimed to validate the gene signature in a separate bariatric surgery cohort and observed a reduced, yet still predictive AUC for successful weight loss based on the three genes ADAP2, SUMO3, and PRKG2. Mechanistically, SUMO3 is involved in the posttranslation modification of target proteins known as SUMOylation [57]. Evidence suggests that SUMOylation is implicated in the pathogenesis of metabolic disorders such as obesity, insulin resistance, and fatty liver [58]. Accordingly, in a recent study that investigated differences in the proteome between obese and normal weight participants, SUMO3 was identified to be one of the top 10 differentially expressed proteins between the two groups [59]. Additional studies are needed to determine the potential functional implications of the identified genes for obesity.

Whether our results might transfer from the identified candidate mechanism based on altered gene expression to an improved metabolic profile needs to be confirmed in larger studies. We acknowledge that the identified gene expression signature needs to be confirmed in independent cohorts.

Another limitation of the current LIWL study is that we cannot specify individual cell populations contributing to the observed gene expression changes in SAT following LIWL. Immune cells, and in particular tissue‐specific macrophages, may change in numbers during weight loss and in regard to their interaction with adipocytes in obesity‐associated adipose tissue, and promote metabolic diseases [60].

The strengths of the current study include a well‐characterized and well‐matched study population at baseline and a prospective study design. All laboratory measurements were performed according to standard operating protocols, and laboratory technicians were were unaware of the status of the samples. To generate a homogeneous study group, only middle‐aged male Caucasians with MetS were included in this study. Although homogeneity was increased among participants, the results hence cannot be generalized to other ethnic groups, sexes, or individuals without MetS. Despite a relatively small sample size, we show strong translational evidence of our findings, since key results could be validated using independent cross‐sectional studies.

In summary, we identified genes that are differentially expressed in SAT following LIWL, which might play an important role in improved insulin signaling and reduced inflammation. Further studies are needed to investigate the functional role of the identified genes in adipose tissue.

## Author Contributions

B.I. designed the research and edited the manuscript. S.Z. and K.R. conducted the research. S.Z. and R.B. wrote the main manuscript. S.Z., K.R., and A.H. analyzed the data and performed statistical analysis. M.B. and C.W. contributed the LOBB expression data. K.L. and A.K. contributed the LATC expression data. A.G., F.N., and A.H. performed RNA seq and preprocessing of the LOBB samples. H.‐J.M., M.F.S., A.M., M.B., H.N.G.M., M.K., K.L., A.K., A.H., K.B., and J.G.S. contributed valuable advice and edited the manuscript. All authors were involved in writing the article and approval of the submitted version.

## Ethics Statement

The study of the main cohort was approved by the ethics committee at Otto‐von‐Guericke University, Magdeburg, Germany (No. 78/11) and was registered at the German Clinical Trials Register (ICTRP Trial Number: U1111‐1158‐3672, retrospectively registered 07 July 2014). Study protocols of LOBB have been approved by the Ethics Committee of the University of Leipzig (LATC: 265–08, 265‐08‐ff; NCT02208141 and AO: 159–12‐21 052 012 and 017‐12ek and BSC: 159–12‐21 052 012). All donors have been informed of the purpose, risks, and benefits of the biobank. All human investigations were conducted according to the principles expressed in the Declaration of Helsinki.

## Consent

Written informed consent was obtained from all subjects involved in the study.

## Conflicts of Interest

M.B. received honoraria as a consultant and speaker from Amgen, AstraZeneca, Bayer, Boehringer‐Ingelheim, Lilly, Novo Nordisk, Novartis, and Sanofi. All other authors declare no conflicts of interest. The funders had no role in the design of the study; in the collection, analyses, or interpretation of data; in the writing of the manuscript; or in the decision to publish the results.

## Supporting information


**Data S1.** Supporting Information.
