# Supplementary material for: Association of Lifestyle‐Induced Weight Loss With Gene Expression in Subcutaneous Adipose Tissue in Metabolic Syndrome
Source: J Diabetes. 2025 Apr 14;17(4):e70083. doi: 10.1111/1753-0407.70083 (PMC11996622; doi:10.1111/1753-0407.70083)
Supplement: Supplementary file 1 — Data S1. Supporting Information. [file JDB-17-e70083-s001.docx]

**Supplementary data**

**
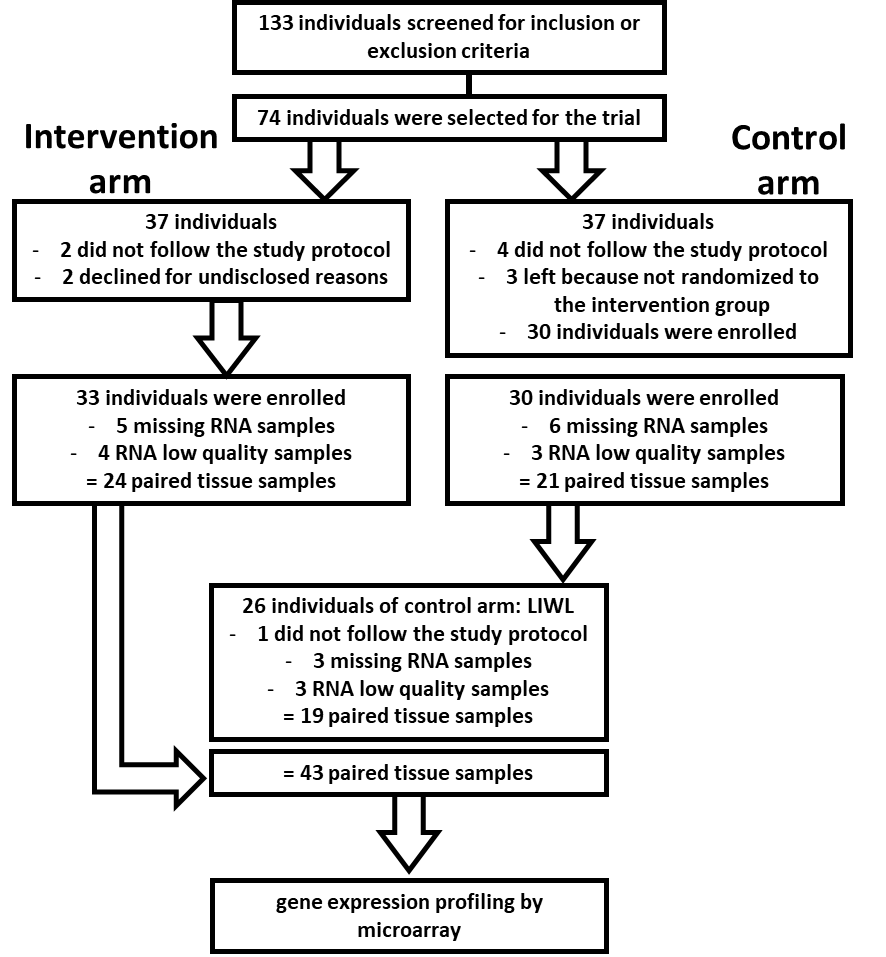
**

**Supplementary Figure 1**: **Schematic study design**. The study is embedded in a two-armed, controlled, monocentric, randomized, 6-month intervention trial. Paired blood samples were collected before and after the 6-months intervention period. Individuals of the control arm were invited during the follow-up period to participate subsequently in the treatment arm. In total, 43 samples were used for unbiased gene expression profiling of SAT.


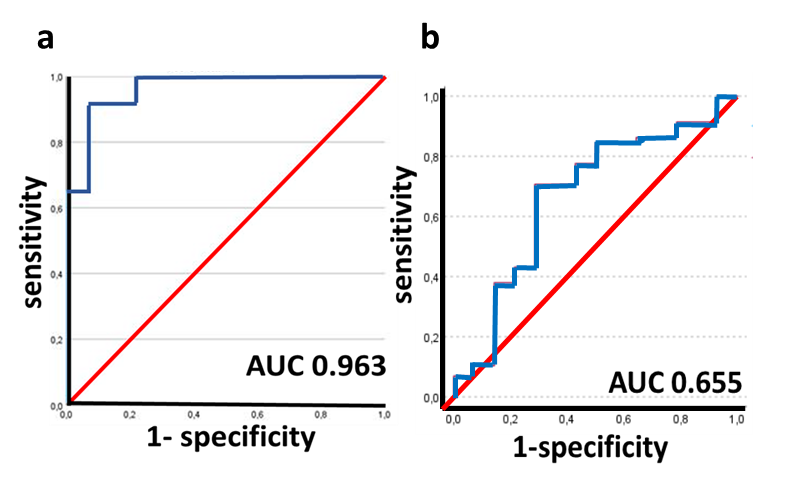


**Supplementary Figure 2**: **ROC curve analysis in an external validation cohort**. Predicted probability (blue line), reference line (red). Gene signature`s AUC was 0.655 (95 % CI: 0.504 – 0.841).


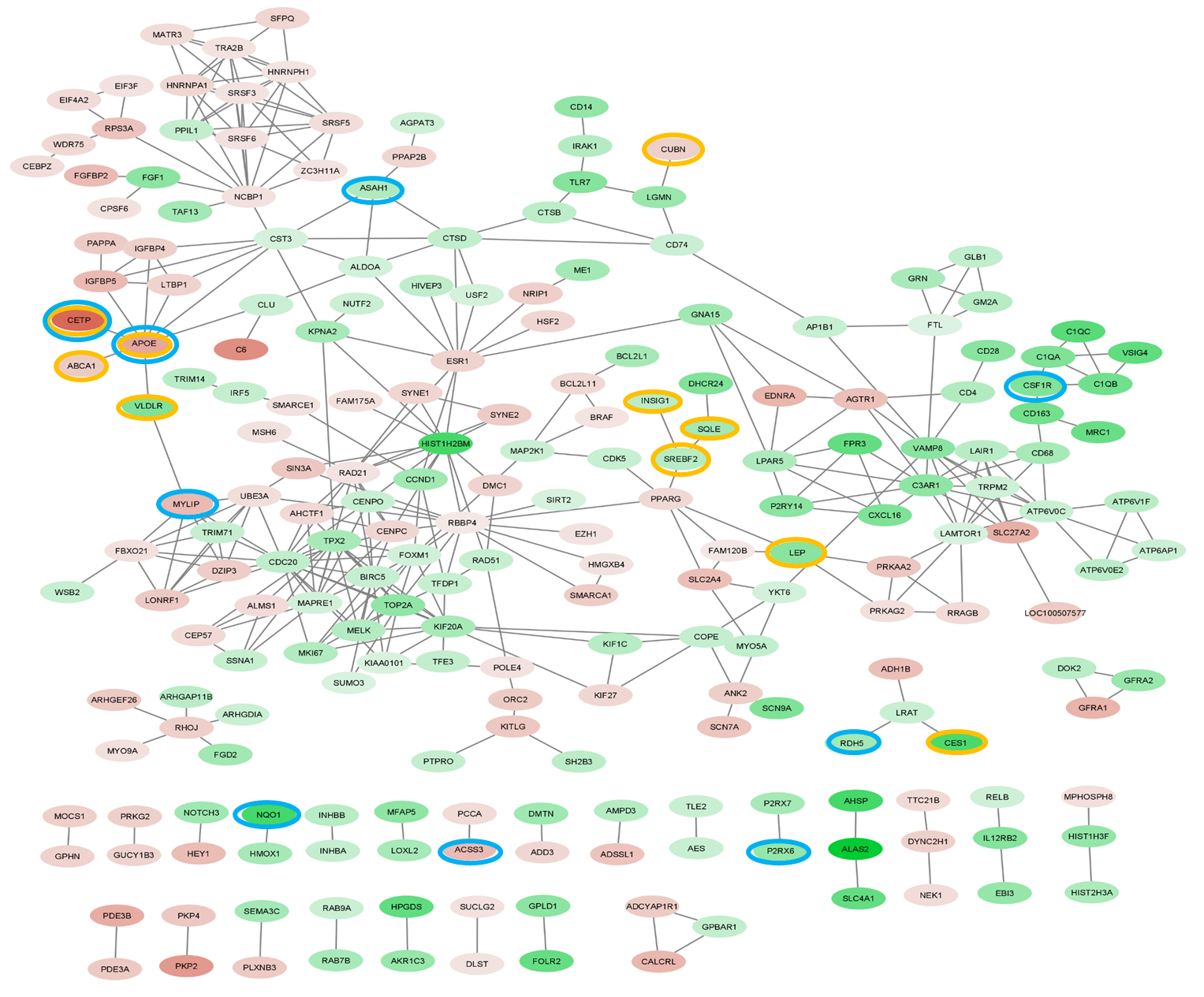


**Supplementary Figure 3.** **Full network created with 642 differentially expressed gene probes as input.** Edges indicate both functional and physical protein associations. Disconnected nodes, and genes not identified in STRING are not shown. Log fold changes are represented in green (downregulated) and red (upregulated), with more intense, darker colors representing lower and higher log fold changes, respectively. Highlighted genes are labeled in colors as determined: genes related to pathway analysis “cholesterol metabolic process” (KEGG, yellow), most significantly DEGs (blue).


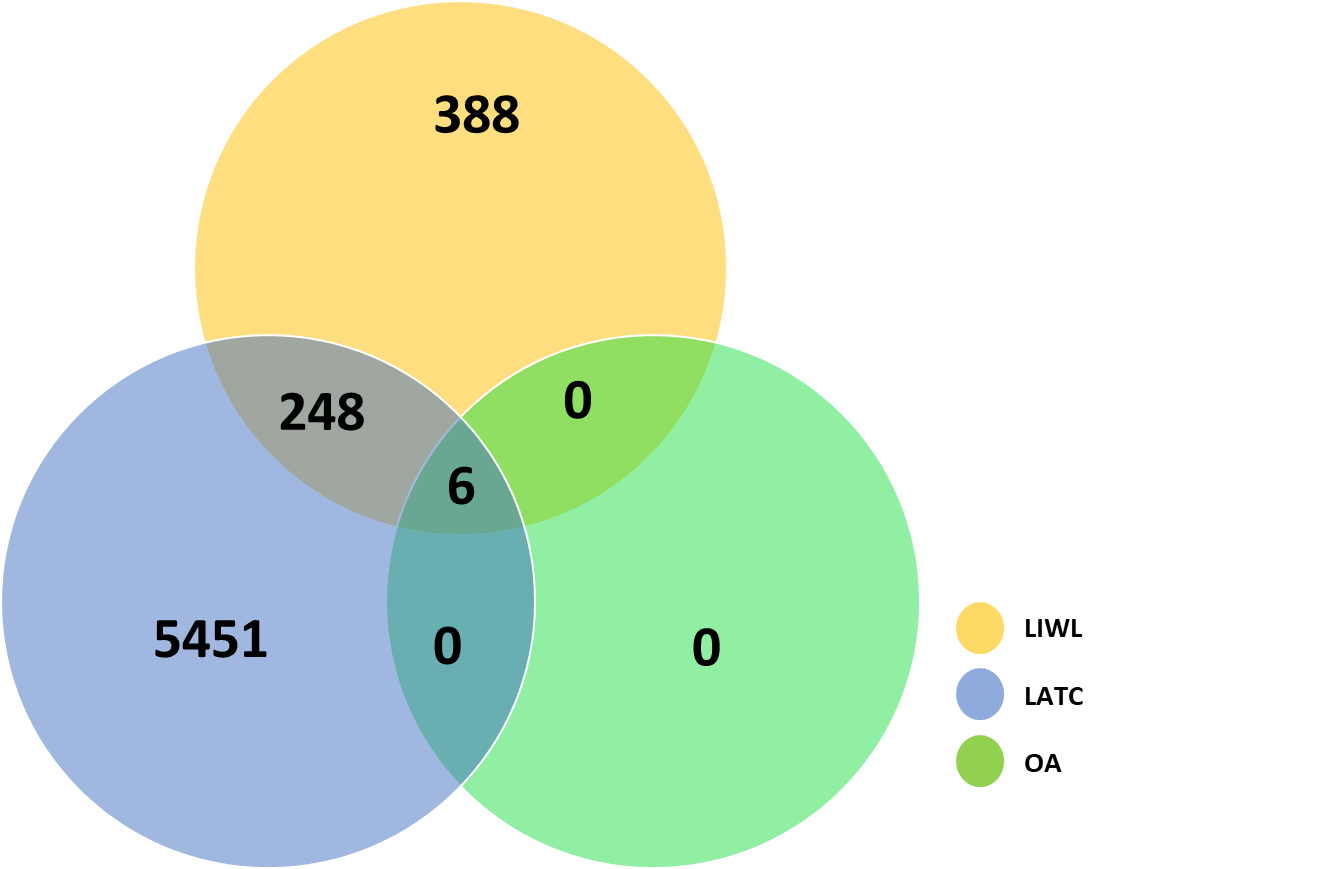


**Supplementary Figure 4**: **Venn diagram showing overlapping DEGs between three cohorts.** Out of 642 DEGs in the LIWL cohort, 254 are overlapping and expressed in the LATC cohort, 6 overlap between the LIWL and the OA cohort and those 6 genes further overlap between all three cohorts. LIWL= lifestyle- induced weight loss, LATC= Leipzig Adipose Tissue Childhood Cohort, OA= adults cohort with or without obesity.


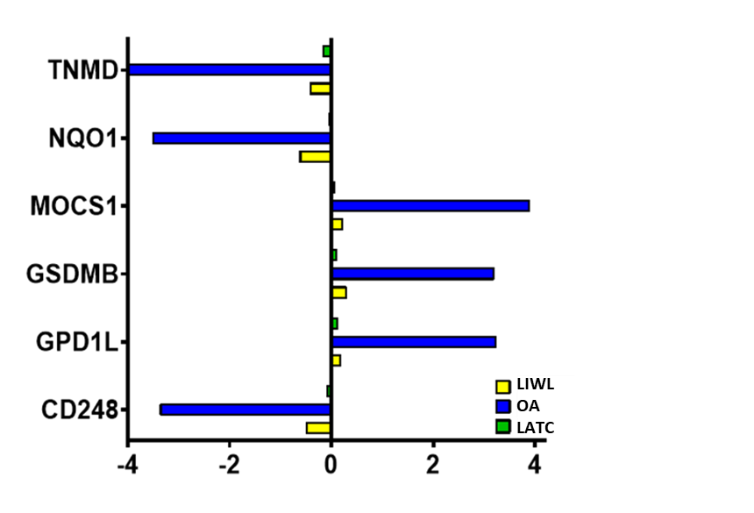


**Supplementary Figure 5**: **Bar graph showing the expression of the six overlapping genes between the three independent cohorts (LATC, LIWL, OA)**. GPD1L= glycerol-3-phosphate dehydrogenase 1 like, GSDMB= gasdermin B, LATC= Leipzig Adipose Tissue Childhood Cohort, LIWL= lifestyle- induced weight loss, MOCS1= molybdenum cofactor synthesis 1, NQO1= NAD(P)H quinone dehydrogenase 1, OA= adults cohort with or without obesity, TNMD= tenomodulin.

**Supplementary Table 1**. Spearman’s correlation coefficient (rho), where *p* value <0.05.

| **GeneID** | **Triglycerides** | **LDLCholest** | **HDLCholest** | **TCholest** |
| --- | --- | --- | --- | --- |
| AASS |  |  |  |  |
| ABCA1 |  |  |  |  |
| ABCB11 | -0.32 |  |  |  |
| AC011891_5 |  |  |  |  |
| ACP5 |  |  |  |  |
| ACSS3 |  |  |  |  |
| ADAM12 | 0.33 |  |  |  |
| ADAM12 | 0.33 |  |  |  |
| ADAM12 | 0.33 |  |  |  |
| ADAP2 |  |  |  |  |
| ADCYAP1R1 |  |  |  |  |
| ADSSL1 |  |  |  |  |
| AKR1C3 | 0.45 |  | -0.31 |  |
| AKR1CL1 | -0.34 |  |  |  |
| ALMS1 |  |  |  | 0.32 |
| AMPD3 |  |  | -0.32 |  |
| AOC2 |  |  |  |  |
| APOE |  |  |  |  |
| ARHGAP11B | 0.41 |  |  |  |
| ARHGEF26 |  |  |  |  |
| ASAH1 | 0.47 |  |  |  |
| ASCC2 |  |  | -0.42 |  |
| ATP6V1F |  |  |  |  |
| ATP6V1G2_DDX39B |  |  |  |  |
| BAG3 |  |  |  |  |
| BCL7B | 0.31 |  |  |  |
| BLNK |  |  |  |  |
| C19orf73 | 0.32 |  |  |  |
| C1QA |  |  |  |  |
| C1QB |  |  |  |  |
| C1QC |  |  |  |  |
| C3AR1 |  |  |  |  |
| C6 |  |  | 0.34 |  |
| CA3 |  |  |  |  |
| CD14 |  |  |  |  |
| CD163 |  |  |  |  |
| CD163L1 |  |  |  |  |
| CD209 |  |  |  |  |
| CD28 |  |  |  |  |
| CD4 |  |  |  |  |
| CD68 |  |  |  |  |
| CD84 |  |  |  |  |
| CDK5 |  |  |  |  |
| CES1 | 0.51 |  | -0.32 |  |
| CETP |  |  |  |  |
| CHD6 |  |  |  |  |
| CLN6 |  |  |  |  |
| CPXM1 |  |  |  |  |
| CREB3L1 |  |  |  |  |
| CSF1R |  |  |  |  |
| CSRP2 |  |  |  |  |
| CTB_111F10_1 |  |  |  |  |
| CTSB |  |  |  |  |
| CUBN |  |  |  |  |
| CXCL16 |  |  |  |  |
| DAPK2 |  |  |  |  |
| DHCR24 |  |  |  |  |
| DIAPH3 |  |  |  |  |
| DMTN |  |  | -0.32 |  |
| DUSP10 | 0.31 |  |  |  |
| EDNRA |  |  |  |  |
| EIF2B5_IT1 |  | -0.31 |  | -0.34 |
| EMB | 0.33 |  |  |  |
| EMC3_AS1 |  |  |  |  |
| EMCN |  |  |  |  |
| ENPP2 |  |  |  |  |
| EPB41L4B |  |  |  |  |
| EPDR1 | 0.31 |  |  |  |
| ESR1 |  |  |  |  |
| FAM198B | 0.31 |  |  | 0.3 |
| FCGR2B |  |  |  |  |
| FCGRT |  | -0.32 |  |  |
| FGF1 |  |  |  |  |
| FHOD3 |  |  |  |  |
| FMN1 |  |  |  |  |
| FOLR2 |  |  |  |  |
| FPR3 |  |  |  |  |
| FTL |  |  |  |  |
| FZD1 |  |  |  |  |
| GABRB2 |  | -0.34 |  |  |
| GCNT1 |  |  |  |  |
| GFRA1 |  |  |  |  |
| GGTA1P |  | -0.38 | -0.33 |  |
| GLB1 |  |  |  |  |
| GNA15 | 0.31 |  | -0.3 |  |
| GPD1L |  |  |  |  |
| GPHN |  |  |  |  |
| GPLD1 | 0.33 |  |  |  |
| GPR34 |  |  |  |  |
| GPX1 |  |  | -0.38 |  |
| GRIN2B | 0.49 |  |  |  |
| GSDMB |  |  |  |  |
| HAMP |  | 0.31 |  |  |
| HAVCR2 | 0.31 |  |  |  |
| HIST1H2BM |  |  |  |  |
| HIST2H3A | 0.38 |  |  |  |
| HIST2H3A | 0.38 |  |  |  |
| HLF |  |  |  |  |
| HMGN2P15 |  |  |  | 0.4 |
| HNRNPH1 | -0.33 |  |  |  |
| HPGDS |  |  |  |  |
| IGFBP4 | -0.34 |  | 0.32 |  |
| IGSF21 |  |  |  |  |
| IL10RA |  |  |  |  |
| IL12RB2 |  |  |  |  |
| IRF5 |  |  |  |  |
| KB_1507C5_4 |  | -0.34 |  |  |
| KDM5D |  |  |  |  |
| KIAA0101 |  |  | 0.34 |  |
| KIF20A |  |  |  |  |
| KIZ |  |  | 0.32 |  |
| KPNA2 |  |  |  |  |
| LARGE |  |  |  |  |
| LEP | 0.36 |  | -0.33 |  |
| LGI2 |  |  | -0.31 |  |
| LGI4 |  |  |  |  |
| LILRB5 |  |  |  |  |
| LINC00152 |  |  |  |  |
| LINC00184 |  | -0.32 | -0.43 |  |
| LINC01070 |  | -0.34 |  |  |
| LINC01094 |  |  |  |  |
| LINC01094 |  |  |  |  |
| LINC01150 | 0.34 |  |  |  |
| LOC100996662 |  |  |  |  |
| LOC101060264 |  |  |  |  |
| LOC101928199 |  |  | -0.34 |  |
| LOC101928803 |  |  |  | 0.33 |
| LOC101929331 |  |  |  |  |
| LOC101929335 |  |  | 0.35 |  |
| LOC102723350 |  |  |  |  |
| LOC102723690 | 0.37 |  |  |  |
| LOC102723825 |  |  |  |  |
| LOC344887 | 0.34 |  |  |  |
| LPAR5 |  |  |  |  |
| LUC7L3 |  |  |  |  |
| M1AP |  | -0.41 |  | -0.31 |
| MAF |  |  | -0.33 |  |
| MAPRE1 | 0.38 |  |  |  |
| MCOLN1 |  |  | -0.36 |  |
| MED14OS |  |  |  |  |
| MELK | 0.33 |  |  | 0.34 |
| MET |  |  |  |  |
| MFAP5 | 0.38 |  |  |  |
| MFF |  |  | 0.37 | 0.33 |
| MFSD5 | 0.32 |  |  |  |
| MKI67 |  |  |  |  |
| MOCS1 |  |  |  |  |
| MRC1 |  |  |  |  |
| MRC1 |  |  |  |  |
| MRPS31P5 |  |  |  |  |
| MS4A4A |  |  |  |  |
| MS4A7 |  |  |  |  |
| MTSS1 |  |  |  |  |
| MYCBP2_AS1 | -0.31 |  |  |  |
| MYLIP |  |  |  |  |
| MYO5A |  |  |  |  |
| NBEA |  |  | 0.3 | 0.39 |
| NCAM2 | -0.46 |  |  | -0.41 |
| NCKAP1L |  |  |  |  |
| NDRG4 | -0.38 |  |  |  |
| NQO1 | 0.37 |  |  |  |
| NRIP1 |  |  |  |  |
| OR2W3 |  |  | -0.31 |  |
| ORMDL2 | 0.32 |  |  |  |
| P2RX6 |  |  |  |  |
| P2RX6P |  |  |  |  |
| P2RX7 |  |  |  |  |
| P2RY14 |  |  |  |  |
| PAPPA |  |  |  |  |
| PDE3B |  |  | 0.3 |  |
| PGM5_AS1 |  |  | 0.36 | 0.38 |
| PKP2 |  |  |  |  |
| PLAGL1 |  |  |  |  |
| PLEK2 |  |  |  |  |
| PLXNB3 |  |  |  |  |
| POM121L9P |  |  |  |  |
| PRG4 |  |  |  |  |
| PTPRO |  |  |  |  |
| RAB7B | 0.31 |  |  |  |
| RAB9A | 0.37 |  | -0.32 |  |
| RALGAPA2 |  |  |  |  |
| RAMP2_AS1 |  |  | 0.39 |  |
| RBBP4 |  |  | -0.3 |  |
| RDH5 | 0.31 |  | -0.33 |  |
| RHOJ |  |  |  |  |
| RHPN2 |  |  |  |  |
| RNASE1 |  |  |  |  |
| RNU6_532P |  |  | 0.3 |  |
| RP1_66C13_3 |  |  |  |  |
| RP1_66C13_3 |  |  |  |  |
| RP11_307L3_4 | -0.33 | -0.35 |  | -0.45 |
| RP11_343N15_2 | 0.41 |  |  |  |
| RP11_508M8_1 | 0.32 |  |  |  |
| RP11_513D5_5 |  |  | -0.35 |  |
| RP3_523C21_2 |  |  |  |  |
| RP4_755D9_1 | 0.33 |  |  |  |
| RP5_1096J16_1 |  |  |  |  |
| S100A11 |  |  |  |  |
| SCAMP2 |  |  |  |  |
| SCIN |  |  |  |  |
| SCN9A |  | -0.31 |  |  |
| SEC23B |  |  |  |  |
| SERF2 | 0.39 |  | -0.36 |  |
| SERINC2 | 0.3 |  |  |  |
| SESN1 |  |  |  |  |
| SFRP4 | 0.47 |  |  |  |
| SH2D4A | 0.32 |  |  |  |
| SIGLEC1 |  | -0.31 |  |  |
| SLAMF8 |  |  |  |  |
| SLC27A2 |  |  |  |  |
| SLC2A4 |  |  |  |  |
| SLC31A2 |  |  |  |  |
| SLC36A1 |  |  |  |  |
| SLC37A2 |  |  |  |  |
| SLC4A1 |  |  | -0.37 |  |
| SLCO2B1 |  |  |  |  |
| SMARCA1 |  |  |  |  |
| SMOC1 | -0.37 |  |  |  |
| SNORA60 |  |  |  |  |
| SRPX |  |  |  |  |
| STON1_GTF2A1L |  |  |  |  |
| STOX1 | -0.3 |  |  |  |
| SUMO3 |  |  | 0.38 |  |
| SVIL |  |  |  |  |
| SVILP1 |  |  |  |  |
| SYNE1 |  |  |  |  |
| SYNPO | 0.3 |  |  |  |
| TAF13 | 0.32 |  |  |  |
| TFEC |  |  |  |  |
| TGFBI |  |  |  |  |
| TGIF2 |  |  |  |  |
| TLR7 |  |  |  |  |
| TMEM63B | 0.39 |  |  |  |
| TNMD |  |  |  |  |
| TOP2A | 0.35 |  |  |  |
| TPST2 | 0.41 |  |  |  |
| TPX2 | 0.48 |  |  |  |
| TUB |  |  |  |  |
| UCHL1 | 0.39 |  |  |  |
| UCP2 |  |  |  |  |
| UST |  |  | 0.36 |  |
| VAMP8 |  |  |  |  |
| VOPP1 | 0.39 |  |  |  |
| VSIG4 |  |  |  |  |
| YKT6 |  |  | -0.38 |  |
| ZNF280D |  |  |  | 0.31 |
| ZNF385A |  |  |  |  |
| ZNF807 |  |  |  |  |

**Supplementary Table 2**

| **ProbeID**  **Top PC2 genes** | **Gene** | **Loading** |
| --- | --- | --- |
| 16908251 | TNS1 | 1.53781E+14 |
| 16887898 | H3F3A | -1.49215E+14 |
| 16695437 | CD48 | -1.48661E+14 |
| 16747594 | ATN1 | 1.45461E+14 |
| 16761176 | KLRB1 | -1.45387E+14 |
| 16787208 | GPR65 | -1.4431E+14 |
| 16879721 | EPAS1 | 1.43032E+14 |
| 16856887 | GNA11 | 1.42638E+13 |
| 16716795 | SORBS1 | 1.42387E+14 |
| 16951917 | CMTM6 | -1.42025E+14 |
| 17089371 | STXBP1 | 1.41936E+14 |
| 16796938 | CDC42BPB | 1.41629E+13 |
| 17046135 | EGFR | 1.41342E+13 |
| 16843167 | EVI2A | -1.40165E+14 |
| 16880122 | SPTBN1 | 1.4011E+13 |
| 16713187 | NRP1 | 1.4009E+14 |
| 16710481 | DOCK1 | 1.4008E+14 |
| 16858805 | NFIX | 1.39997E+14 |
| 16705064 | UBE2D1 | -1.39668E+14 |
| 16856946 | NFIC | 1.39363E+14 |
| 16882162 | TCF7L1 | 1.39291E+14 |
| 16801707 | TLN2 | 1.39017E+14 |
| 16711909 | FRMD4A | 1.38871E+14 |
| 16863652 | EHD2 | 1.38847E+14 |
| 16714405 | RP11-556E13.1 | -1.38695E+14 |
| 16975419 | APBB2 | 1.38626E+14 |
| 16731967 | PHLDB1 | 1.38521E+14 |
| 17005871 | HIST1H2BN | -1.38389E+14 |
| 16985440 | MAST4 | 1.37913E+14 |
| 16858312 | C19orf38 | -1.37826E+14 |
| 16692624 | HIST2H2BC | -1.37779E+14 |
| 17035441 | AIF1 | -1.375E+14 |
| 16683105 | HSPG2 | 1.37474E+13 |
| 17114970 | GABRE | 1.37071E+13 |
| 16711037 | DIP2C | 1.36277E+14 |
| 16955800 | PRICKLE2 | 1.36017E+14 |
| 16707955 | FRAT1 | -1.35997E+14 |
| 17030643 | AIF1 | -1.35356E+14 |
| 16940172 | CCR2 | -1.35268E+14 |
| 16699174 | PTPN14 | 1.35194E+14 |
| 17006683 | AIF1 | -1.3513E+14 |
| 17027817 | AIF1 | -1.3513E+14 |
| 17038140 | AIF1 | -1.3513E+14 |
| 17040735 | AIF1 | -1.3513E+14 |
| 16690789 | DENND2D | -1.34807E+14 |
| 17120140 | NRBF2 | -1.34693E+14 |
| 16752715 | LRP1 | 1.34633E+14 |
| 17030627 | LST1 | -1.34414E+14 |
| 17035425 | LST1 | -1.34414E+14 |
| 16886860 | LOC643072 | -1.34407E+14 |
| 17026272 | LST1 | -1.34354E+14 |
| 16919265 | ZHX3 | 1.34237E+13 |
| 17002612 | SLIT3 | 1.34234E+14 |
| 16845158 | PTRF | 1.34212E+14 |
| 16812344 | BCL2A1 | -1.34164E+14 |
| 17006666 | LST1 | -1.3415E+14 |
| 16867326 | PLIN4 | 1.33926E+14 |
| 16854202 | ABHD3 | -1.3387E+13 |
| 16834516 | AOC3 | 1.33857E+13 |
| 16914733 | ZFAS1 | -1.33665E+14 |
| 17040719 | LST1 | -1.3352E+14 |
| 16991385 | LARP1 | 1.33458E+14 |
| 16867432 | PTPRS | 1.33437E+14 |
| 16761726 | PLBD1 | -1.33388E+14 |
| 17106997 | MST4 | -1.3338E+14 |
| 16674805 | LAMC1 | 1.33325E+12 |
| 16857467 | TRIP10 | 1.33259E+14 |
| 17110463 | CFP | -1.33155E+14 |
| 16984801 | GZMA | -1.3314E+14 |
| 16876849 | ASAP2 | 1.33124E+14 |
| 16831562 | MPRIP | 1.33061E+14 |
| 16825539 | C16orf54 | -1.32903E+14 |
| 17027801 | LST1 | -1.32867E+14 |
| 17033344 | LST1 | -1.32867E+14 |
| 17101537 | TLR8 | -1.32832E+14 |
| 16745016 | CD3D | -1.32741E+14 |
| 16670377 | HIST2H4B | -1.32741E+14 |
| 16692626 | HIST2H4B | -1.32741E+14 |
| 17038124 | LST1 | -1.32634E+14 |
| 17092794 | PTPLAD2 | -1.32452E+14 |
| 16922011 | BACH1 | -1.3243E+14 |
| 16929442 | TIMP3 | 1.32391E+14 |
| 17105076 | P2RY10 | -1.32322E+14 |
| 16893846 | PXDN | 1.32288E+14 |
| 16953944 | LAMB2 | 1.32275E+14 |
| 16819827 | CMTM1 | -1.32266E+14 |
| 17012767 | TBPL1 | -1.32117E+14 |
| 16730268 | AMOTL1 | 1.31999E+13 |
| 16874945 | FPR1 | -1.31813E+14 |
| 16832049 | EPN2 | 1.3173E+14 |
| 17025177 | DYNLT1 | -1.31647E+14 |
| 16829158 | RNF166 | -1.314E+12 |
| 16751601 | TENC1 | 1.31256E+14 |
| 16847578 | LIMD2 | -1.31167E+14 |
| 16782072 | TRAJ26 | -1.31155E+14 |
| 16953241 | MAP4 | 1.31136E+14 |
| 17095375 | AGTPBP1 | -1.31126E+14 |
| 17057422 | SNORA9 | -1.30797E+14 |
| 16669058 | RP4-666F24.3 | -1.30641E+14 |
| 17045005 | FKBP9 | 1.30602E+14 |

| **ProbeID**  **Top PC3 genes** | **Gene** | **Loading** |
| --- | --- | --- |
| 16749511 | MRPS35 | 1.4339E+14 |
| 17079436 | HRSP12 | 1.43376E+14 |
| 16803533 | HYKK | 1.42377E+14 |
| 16745041 | IFT46 | 1.42323E+14 |
| 16662744 | NDUFS5 | 1.40739E+14 |
| 16698656 | EIF2D | 1.40244E+14 |
| 17023308 | HDDC2 | 1.3888E+13 |
| 16729444 | AAMDC | 1.37569E+14 |
| 17062000 | LINC00998 | 1.37522E+14 |
| 16731105 | C11orf1 | 1.37017E+13 |
| 16671503 | CKS1B | 1.36317E+14 |
| 16866867 | MOB3A | -1.36213E+14 |
| 17009620 | TMEM14A | 1.3537E+14 |
| 17112956 | TCEAL8 | 1.33962E+14 |
| 16927040 | MRPL40 | 1.33951E+14 |
| 16692772 | ADAMTSL4-AS1 | -1.33894E+14 |
| 16782068 | TRAJ28 | -1.33572E+14 |
| 16878122 | ATRAID | 1.33471E+14 |
| 16719802 | ADAM8 | -1.3334E+14 |
| 17121830 | AMZ2P1 | 1.33201E+13 |
| 16716327 | RNLS | 1.33093E+14 |
| 16913936 | IFT52 | 1.32108E+14 |
| 16860746 | UBA2 | 1.31905E+12 |
| 16977912 | PIGY | 1.31847E+14 |
| 16848902 | UNC13D | -1.31506E+14 |
| 16774669 | NUDT15 | 1.31156E+14 |
| 16777634 | MTIF3 | 1.30522E+14 |
| 16786713 | IFT43 | 1.29729E+14 |
| 16795826 | CCDC88C | -1.29428E+14 |
| 17029594 | AGER | -1.27833E+13 |
| 17034585 | AGER | -1.27833E+13 |
| 17039794 | AGER | -1.27833E+13 |
| 17042290 | AGER | -1.27833E+13 |
| 16914169 | NA | -1.27701E+14 |
| 16723748 | C11orf74 | 1.27685E+14 |
| 17056211 | HIBADH | 1.27099E+14 |
| 17024753 | RMND1 | 1.26974E+14 |
| 16678710 | GNPAT | 1.26892E+14 |
| 16740797 | MRPL11 | 1.26723E+13 |
| 16784947 | MNAT1 | 1.26593E+14 |
| 16819355 | NLRC5 | -1.26282E+13 |
| 16879276 | MORN2 | 1.26262E+14 |
| 17009607 | TRAM2-AS1 | 1.26044E+14 |
| 16940260 | NBEAL2 | -1.26024E+14 |
| 16756995 | TCTN1 | 1.25942E+14 |
| 16889209 | C2orf47 | 1.25584E+14 |
| 16793460 | TIMM9 | 1.24803E+14 |
| 16791898 | CFL2 | 1.24658E+14 |
| 16766658 | AGAP2 | -1.24545E+14 |
| 16985629 | MRPS36 | 1.24108E+14 |
| 16701077 | FH | 1.24065E+14 |
| 16948087 | NAALADL2 | 1.24025E+14 |
| 16995957 | PAIP1 | 1.2394E+14 |
| 16794260 | NA | 1.23729E+14 |
| 16780640 | NA | 1.23646E+14 |
| 16746437 | THYN1 | 1.23613E+14 |
| 16959187 | MRPL3 | 1.23562E+14 |
| 17064185 | ZNF746 | -1.23491E+14 |
| 16968407 | COPS4 | 1.23388E+14 |
| 16664150 | TMEM69 | 1.23262E+14 |
| 17026994 | AGER | -1.23189E+14 |
| 17037086 | AGER | -1.23189E+14 |
| 16701407 | TFB2M | 1.23129E+14 |
| 16902221 | C2orf76 | 1.23121E+12 |
| 16677153 | LINC00467 | 1.2309E+13 |
| 16764131 | PRKAG1 | 1.23076E+14 |

| **ProbeID**  **Top PC4 genes** | **Gene** | **Loading** |
| --- | --- | --- |
| 16858991 | PKN1 | -1.83382E+14 |
| 16842027 | FLII | -1.78545E+14 |
| 16694804 | ARHGEF11 | -1.72875E+14 |
| 16667155 | SNORD21 | 1.65333E+14 |
| 16822728 | CLCN7 | -1.64691E+12 |
| 16856910 | NCLN | -1.60992E+13 |
| 16807315 | NA | 1.59085E+14 |
| 16808314 | PPIP5K1 | -1.56991E+14 |
| 16689227 | SEP15 | 1.56625E+14 |
| 17123916 | CTBP2P4 | -1.56435E+14 |
| 17103451 | HDAC6 | -1.5566E+14 |
| 16817521 | SPNS1 | -1.55183E+14 |
| 16706082 | SEC24C | -1.54883E+14 |
| 16900022 | KRCC1 | 1.53985E+14 |
| 16956661 | CLDND1 | 1.53647E+14 |
| 17124598 | DTX2 | -1.53513E+14 |
| 16969988 | RNU1-138P | -1.53509E+14 |
| 16660906 | TMEM50A | 1.53114E+14 |
| 17047258 | GATSL2 | -1.53102E+14 |
| 16940362 | PTPN23 | -1.52883E+13 |
| 17122888 | NA | 1.52807E+14 |
| 17091571 | MAN1B1 | -1.51984E+14 |
| 17017196 | NA | 1.51935E+14 |
| 16838855 | HGS | -1.51634E+14 |
| 17115394 | HCFC1 | -1.51495E+14 |
| 16815001 | TRAF7 | -1.50726E+14 |
| 16694361 | ARHGEF2 | -1.50658E+14 |
| 17121682 | RP11-927P21.11 | -1.50582E+14 |
| 16936452 | PLXNB2 | -1.50519E+14 |
| 16852497 | WDR7 | -1.50222E+14 |
| 16858823 | NACC1 | -1.50192E+14 |
| 17004335 | NA | -1.49592E+14 |
| 16867378 | DPP9 | -1.49507E+14 |
| 16781695 | ARHGEF40 | -1.48862E+13 |
| 16678105 | SRP9 | 1.48489E+14 |
| 17036605 | BAG6 | -1.48069E+14 |
| 17115505 | FLNA | -1.47653E+13 |
| 16857254 | FEM1A | -1.47647E+14 |
| 16808316 | PPIP5K1 | -1.47383E+14 |
| 16829242 | GALNS | -1.469E+14 |
| 16890314 | RPE | -1.4638E+14 |
| 16860017 | GATAD2A | -1.46296E+14 |
| 16973375 | GAK | -1.45894E+14 |
| 16794257 | NA | 1.45879E+14 |
| 17093260 | NOL6 | -1.45866E+14 |
| 16651653 | NA | 1.45851E+14 |
| 16981657 | NA | 1.45502E+14 |
| 17017244 | BAG6 | -1.45119E+14 |
| 17031887 | BAG6 | -1.45119E+14 |
| 17034357 | BAG6 | -1.45119E+14 |
| 16912140 | ENTPD6 | -1.45033E+14 |
| 16831442 | TRPV2 | -1.44249E+14 |
| 16683925 | SLC9A1 | -1.44201E+14 |
| 16790262 | NA | 1.44098E+14 |
| 16936535 | SBF1 | -1.44001E+14 |
| 17122060 | RPL10 | 1.43999E+14 |
| 16788713 | MIR411 | -1.43614E+14 |
| 16920287 | DPM1 | 1.43567E+14 |
| 17022355 | NA | 1.43459E+14 |
| 16914374 | PCIF1 | -1.43273E+14 |
| 16971421 | NA | 1.43182E+14 |
| 16804942 | FURIN | -1.43179E+14 |
| 16799707 | VPS18 | -1.43173E+14 |
| 16711456 | NA | 1.43146E+14 |
| 16827850 | VAC14 | -1.43111E+14 |
| 16706135 | ZSWIM8 | -1.43092E+14 |
| 16733589 | RNU6ATAC12P | -1.42977E+14 |
| 16823098 | NA | -1.42929E+14 |
| 16810964 | NA | 1.42689E+14 |
| 16711444 | NA | 1.42302E+14 |
| 16908612 | NA | -1.42132E+14 |
| 16855492 | NA | 1.42054E+14 |
| 17029102 | BAG6 | -1.41914E+14 |
| 17039400 | BAG6 | -1.41914E+14 |
| 17041883 | BAG6 | -1.41914E+14 |
| 16900519 | NA | -1.41556E+14 |
| 16899619 | TGOLN2 | -1.41145E+12 |
| 16977352 | NA | 1.41001E+13 |
| 16684385 | SNORD103A | -1.41E+13 |
| 16684387 | SNORD103A | -1.41E+13 |
| 16740814 | ZDHHC24 | -1.40751E+14 |
| 16853277 | NFATC1 | -1.40698E+14 |
| 17099980 | SEC16A | -1.4065E+14 |
| 16940738 | APEH | -1.40558E+14 |
| 16682348 | ATP13A2 | -1.40378E+13 |
| 16748453 | LOH12CR1 | -1.40335E+14 |
| 16752908 | MARS | -1.39984E+14 |
| 17032011 | DDAH2 | -1.39905E+14 |
| 17034481 | DDAH2 | -1.39905E+14 |
| 17036729 | DDAH2 | -1.39905E+14 |
| 17042007 | DDAH2 | -1.39905E+14 |
| 16824127 | LOC100288162 | -1.39819E+14 |
| 16738869 | PRPF19 | -1.39801E+14 |
| 17039524 | DDAH2 | -1.39625E+14 |
| 16926527 | POFUT2 | -1.39613E+14 |
| 16823991 | RSL1D1 | 1.39165E+14 |
| 16889759 | NA | 1.39136E+14 |
| 16722391 | NA | 1.39045E+14 |
| 16782786 | NOP9 | -1.38975E+14 |
| 17089606 | SLC27A4 | -1.38918E+14 |
